# Treatment of Carcinoma Uteri Cases Not Justifiably Treated by Radical Operation

**Published:** 1904-06

**Authors:** 


### Treatment of Carcinoma Uteri Cases Not Justifiably Treated by Radical Operation.

THE first duty in a hopeless case of this disease is to relieve suffering. Opiates, according to Tuttle (*Boston Medical and Surgical Journal,* April 17, 1902,) should be used judiciously to combat the pain and the irritation. Small doses should be given; to obtain the longest continued effect from a given dose, it should be administered by the rectum.

Cauliflower growths and painful granulations may be kept down by surgical means and local treatment. In a similar manner the secretions may be purified, autoinfections prevented, and the irritation of neighboring parts that have been soiled by the acrid discharges may be allayed. The local treatment is to maintain as far as possible a clean granulating condition of the ulcerated surface.

Hemorrhages are practically treated with the curette and cautery. It may be necessary, rarely, to resort to packing or to chemical hemostatics.

For the complicated and distressing bladder conditions that sometimes arise, about all that can be done is to keep the patient half-way dry, grease the skin and parts exposed to irritation with a soothing ointment, and to administer opiates sufficient to control or to modify the pain. The bowels, in rectal complications, should be kept open with saline cathartics.

As the disease extends into the vaginal region, the marked infiltration may be treated with cleansing solutions, hot fomentations, and bland unguents.

Hypodermic injections of antiseptic fluids, as a method of treatment, is looked on with favor by Tuttle, who is inclined to a theory that cancer is the result of a change in the environments of a cell or group of cells, by which their normal nutrition is affected, and which results in their active proliferation, contrary to the natural law of growth. If this theory be accepted, he looks upon the individual cells making up the neoplasm as foreign bodies, and holds them singly in the same relation to the body as foreign elements introduced from without, such as bacterial or ameboid forms of life, and he suggests a hypodermic treatment with various chemicals, such as oils, alcohols, and other agencies.—*Therapeutic Gazette.*

